# Industry-Sponsored Research: A More Comprehensive Alternative

**DOI:** 10.1371/journal.pmed.0030463

**Published:** 2006-10-31

**Authors:** Peter Mansfield

Julio Sotelo's proposal for pharmaceutical research to be organised by a Collegiate Research Council (CRC) funded by drug companies [1] is one of several alternatives that deserve debate [2].

The Sotelo proposal has advantages, but if the CRC is a single international monopoly how could the risk of corruption and inefficiency be managed? Alternatively, if there were competing CRCs, they would be under pressure to compromise to win more contracts, as happens already with contract research organisations.

Fiona Godlee has proposed that pharmaceutical manufacturers be banned from researching their products [3]. She suggests that “to get their products licensed [drug companies] would contribute to a central pot for independent, publicly funded clinical trials.” She did not specify what percentage of the “central pot” would be funded by taxpayers versus pharmaceutical companies. If the funding was mostly from pharmaceutical companies then her proposal is similar to Sotelo's. If not, how will governments to be persuaded to allocate adequate funds?

My organisation, Healthy Skepticism Inc., advocates a more comprehensive alternative that will also reduce the harms currently caused by misleading promotion, biased industry funding of education, and high drug prices. Our alternative is politically achievable because implementation can be achieved without increasing costs for pharmaceuticals currently paid by individuals and/or third party payers (governments or insurance companies) whilst securing long-term competitive return on investment for the pharmaceutical industry.

Pharmaceutical companies currently have four main functions: manufacturing, research, promotion, and education. Performance of those functions is currently distorted by incentive systems that reward only activities that increase sales of more expensive drugs regardless of the impact on health care. We recommend that these four functions be paid for separately by government agencies via iterative open competitive public tender. This would allow the relevant divisions and subcontractors of pharmaceutical companies to compete with universities and other non-profit organisations for funding to provide each function separately. Incentives can then to be aligned to reward quality performance at each function separately. If a company performed poorly, e.g., committed research fraud or provided misleading promotion, then it would not get funding for that function in the next tender round. Drug prices would no longer include a premium for research, promotion, and education. Consequently, drug companies would no longer fund those functions from drug sales. Lower prices would make drugs more cost-effective for larger numbers of people.

Our recommendations can be implemented quickly or slowly by gradually reducing prices and transferring the savings to organisations that fund research (e.g., the United Kingdom Medical Research Council); education (e.g., medical schools and specialist colleges); and promotion (e.g., Best Practice Advocacy Centre, New Zealand). We also recommend improving regulation of pharmaceutical companies and improving education, incentive systems, and regulation for health professionals[4–7].

## References

[pmed-0030463-b001] Sotelo J (2006). Regulation of clinical research sponsored by pharmaceutical companies: A proposal. PLoS Med.

[pmed-0030463-b002] Baker D (2004). Financing drug research: What are the issues?. http://www.cepr.net/publications/intellectual_property_2004_09.htm.

[pmed-0030463-b003] Godlee F (2006). Can we tame the monster?. BMJ.

[pmed-0030463-b004] Sweet M (2004). Doctors and drug companies are locked in “vicious circle”. BMJ.

[pmed-0030463-b005] Mansfield PR (2004). Healthy skepticism about drug promotion: Memorandum for the UK House of Commons Health Committee Inquiry: The influence of the pharmaceutical industry. Healthy Skepticism. http://www.healthyskepticism.org/news/issue.php?id=6.

[pmed-0030463-b006] Mansfield P, Rogers W, Jureidini J (2005). Submission from Healthy Skepticism re RACP ethical guidelines. Healthy Skepticism. http://www.healthyskepticism.org/news/issue.php?id=15.

[pmed-0030463-b007] Mansfield PR (2005). Banning all drug promotion is the best option pending major reforms. J Bioeth Inq.

